# Down-Regulation of Acyloxyacyl Hydrolase Expression in Alzheimer’s Disease Impairs LPS Detoxification and Contributes to Brain Pro-Inflammatory Signaling

**DOI:** 10.3390/molecules31030486

**Published:** 2026-01-30

**Authors:** Yuhai Zhao, Nathan M. Sharfman, Vivian R. Jaber, Christopher M. Taylor, Walter J. Lukiw, Nicolas G. Bazan

**Affiliations:** 1 Neuroscience Center of Excellence, School of Medicine, Louisiana State University Health New Orleans, New Orleans, LA 70112, USA; yzhao4@lsuhsc.edu (Y.Z.); nsharf@lsuhsc.edu (N.M.S.); vjaber@lsuhsc.edu (V.R.J.); wlukiw@lsuhsc.edu (W.J.L.); 2 Department of Microbiology, Immunology & Parasitology, School of Medicine, Louisiana State University Health New Orleans, New Orleans, LA 70112, USA; ctay15@lsuhsc.edu

**Keywords:** acyloxyacyl hydrolase (AOAH), Alzheimer’s disease (AD), *Bacteroides fragilis*, hydrolases, inflammatory neurodegeneration, lipases, lipopolysaccharide (LPS), microRNA-450b (miRNA-450b), neuroinflammation

## Abstract

Lipopolysaccharides (LPSs) are potent pro-inflammatory neurotoxins abundant in the gut microbiome and originate primarily from Gram-negative bacteria, such as *Escherichia coli*. LPS levels increase with brain aging and accumulate around neurons in Alzheimer’s disease (AD) brains. Microbiome-generated LPS and other endotoxins cross gut barriers, enter systemic circulation, and translocate across the blood–brain barrier into vascularized brain regions. These processes are exacerbated by aging and neurovascular diseases. Although pro-homeostatic systems mitigate LPS effects, these defenses can fail. This study provides the first evidence that acyloxyacyl hydrolase (AOAH; EC 3.1.1.77), a microglia-enriched LPS detoxifying enzyme, shows reduced expression in AD brain tissue. Analysis of AD patient brains revealed reduced AOAH messenger RNA (mRNA) levels, accompanied by elevated expression of microRNA (hsa-miR-450b-5p), an inflammation regulator. Furthermore, luciferase reporter assays demonstrated that miR-450b-5p specifically targets the AOAH 3′-UTR, leading to a dose-dependent suppression of reporter activity. Also, in vitro experiments on human neuronal glial (HNG) cells further confirmed down-regulation of AOAH expression at protein levels by miR-450b-5p. These findings suggest miR-450b-5p-mediated AOAH deficiency drives LPS-associated neurotoxicity and inflammatory neurodegeneration in AD.

## 1. Introduction

The gastrointestinal (GI) tract in *Homo sapiens*, home to 98% of the human microbiome, contains a remarkably diverse and dynamic population of microbes. These microbes play critical roles in maintaining cellular homeostasis, mediating endocrine signaling, regulating inflammation and immune responses, and supporting digestion, nutrition, growth, and development [1,2].

The GI-tract microbiome primarily comprises bacteria, with microbial eukaryotes, archaea, fungi, protozoa, viruses, and other commensal microorganisms contributing to about 2% or less of its composition [1,2,3]. Quantitatively, the human GI-tract microbiota: (i) forms a complex symbiotic relationship with the host, essential for maintaining health and physiological balance [3,4]; (ii) demonstrates significant variation in abundance, stoichiometry, and aerobic requirements along the entire 10 m length of the GI tract [2]; (iii) includes approximately 10^14^ bacteria representing at least 10^3^ distinct bacterial species, forming a highly interactive microbial ecosystem [5] (iv) undergoes changes in abundance and speciation influenced by developmental stage, lifestyle, diet, aging, and GI-tract disturbances or diseases, including metabolic disorders and Alzheimer’s disease (AD) [6]; and (v) can experience shifts in microbial abundance, composition, stoichiometry, and complexity that reflect a pathological condition termed “dysbiosis,” which may contribute to the development of various progressive, age-related neurological disorders [5,6,7].

Our understanding of the human GI-tract microbiome as a critical contributor to human health and neurological disease has developed relatively recently. Direct scientific evidence linking microbial activities and their secretory products to the onset of neurological diseases in the brain and central nervous system (CNS) remains surprisingly limited [8]. Recent experimental findings, however, have begun to elucidate the connection between microbial-derived neurotoxic exudates, such as lipopolysaccharides (LPSs) from Gram-negative bacteria (e.g., *Escherichia coli*) in the GI tract and neurological disorders [9,10]. Another interesting gut commensal bacteria is the *Bacteroides*, which are important clinical pathogens and are found in most anaerobic infections. One of the species, *B. fragilis*, produces structurally atypical LPS with relatively low intrinsic endotoxin activity; however, its high capacity to liberate endotoxins upon antibiotic exposure may explain its frequent association with severe clinical infections and elevated mortality [11]. Together, these findings indicate that: (i) LPS is abundant in CNS compartments associated with age-related neurodegenerative diseases, including sporadic AD [12]; (ii) LPS contributes to the activation of the pro-inflammatory transcription factor NF-kB (p50/p65) complex and associated signaling pathways [13,14]; and (iii) Upregulated LPS-mediated increases in reactive oxygen species (ROS) and NF-kB (p50/p65) have been directly linked to elevated levels of a specific family of NF-kB-sensitive microRNAs (miRNAs). These miRNAs downregulate their target messenger RNAs (mRNAs), leading to innate-immune deficits, inflammatory signaling, and progressive neuropathological changes in the CNS [14].

Many types of human cells have developed molecular strategies to counter the neurotoxic and pro-inflammatory effects of LPS. One primary cellular defense mechanism involves enzymatic detoxification, facilitated by acyloxyacyl hydrolase (AOAH; EC 3.1.1.77). AOAH is a highly conserved vertebrate host enzyme with multiple activities, including phospholipase, lysophospholipase, diacylglycerol lipase, and acyltransferase functions [15,16,17]. It detoxifies LPS by removing the secondary (acyloxyacyl-linked) fatty acyl chains from the lipid A region, rendering LPS immunologically inert [15]. Secreted AOAH plays a critical role in modulating host inflammatory responses to Gram-negative bacterial invasion and LPS release. AOAH expression has been observed in both neural and myeloid cells [16,17]. This study focuses on investigating AOAH expression in the aging brain and in AD-affected cells and tissues, particularly in scenarios where: (i) LPS accumulate within neurons of the temporal lobe and hippocampus, two anatomical regions heavily impacted by AD [8,12]; (ii) AOAH messenger RNA (mRNA) has been found to be down-regulated in AD [18,19]; and (iii) the absence of AOAH may contribute to LPS-associated neurotoxicity and support progressive inflammatory neurodegeneration in the AD-affected brain, CNS, and other tissues [8,16]. The present study reveals that HNG cells in primary culture transfected with a pLightSwitch 3′-UTR luciferase reporter vector containing the AOAH mRNA 3′ untranslated region (3′-UTR) exhibited down-regulated AOAH expression when treated with *Homo sapien* (hsa)-miR-450b-5p, an inflammation regulator [20]. This suggests: (i) down-regulation of AOAH expression in the brain may be partially mediated by a miR-450b-5p-driven epigenetic mechanism; and (ii) a resultant lack of AOAH in the AD brain may exacerbate susceptibility to the pro-inflammatory and neurotoxic effects of microbiome-derived LPS.

## 2. Results

### 2.1. AOAH and miR-450b-5p Expression in AD

**Figure 1:**
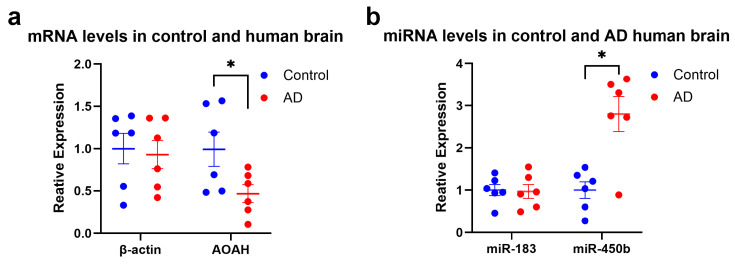
**AOAH mRNA and miR-450b-5p abundance in AD versus age- and gender-matched controls.** Details of brain tissues and quality control parameters have been previously reported in detail (see Table 2 in reference [21]). Briefly control temporal lobe neocortical tissues had an age-range of 66–71 yr; mean +/− 1 SD of 69.0 +/− 1.8 yr, PMI * range of 1.3–3.0 h; N = 6 (3F; 3M) and AD tissues had an age-range of 67–76 yr; mean+/− 1 SD of 70.3 +/− 3.3 yr, PMI range of 1.3–3.0 h; N = 6 (3F; 3M). There were no significant differences in the age, gender or PMI between the AD and control brain groups. No significant differences in the total RNA yields or spectral quality between AD and control groups were noted. (**a**) While there were no significant differences in β-ACTIN mRNA abundance between control and AD, there was an observed decrease in AOAH mRNA to 0.47-fold of controls in AD-affected tissues. (**b**) Similarly, while there were no significant differences in miR-183 control miRNA abundance between control and AD, miRNA quantification indicated an increase in the abundance of miR-450b-5p to greater than 2.8-fold over controls. A strong miR-450b-5p binding site in the human AOAH mRNA 3′-UTR suggested that miR-450b-5p may interact with the AOAH 3′-UTR to drive down-regulation of the expression of that mRNA (Figure 2). Dot plot graphs show data as Mean ± Standard Error of Mean (SEM). Individual data points are shown as dots, middle bars as Means and error bars as SEM. Statistical significance was assessed using one-way ANOVA with Tukey’s post hoc test; * p < 0.05; * (PMI = post-mortem interval = period between brain tissue isolation and freezing at −81 °C).

The LPS-detoxifying enzyme AOAH was readily identified in the temporal lobe neocortex and hippocampus of human brains [18,19]. Analysis of samples from six AD brains and six age- and gender-matched control brains using mRNA qPCR assays (LC Sciences, Houston, TX, USA) revealed reduced levels of AOAH mRNA in the neocortex of AD-affected brains compared to controls (Figure 1a), suggesting decreased AOAH expression in brain cells, including neurons and glia. miR-450b-5p specifically exhibited significantly higher abundance in the neocortex of AD brains compared to age- and gender-matched controls (Figure 1b).

### 2.2. Functional Validation Assay Indicate That miR-450b-5p Targets AOAH 3′UTR Specifically and Dose-Dependently

**Figure 2:**
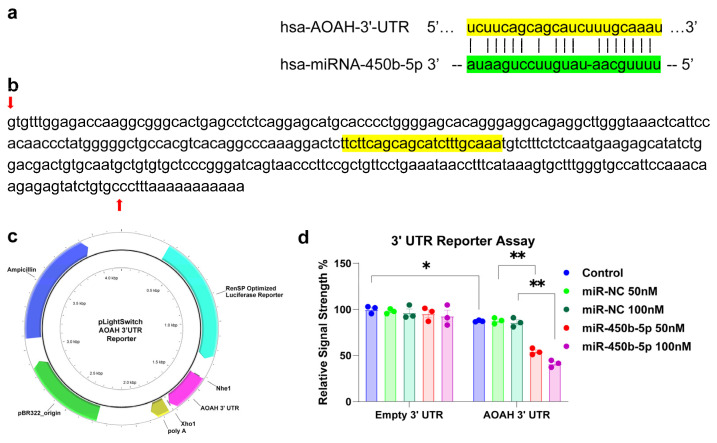
**Acyloxyacyl hydrolase (AOAH) mRNA 3′-UTR interaction with the *Homo sapien* (hsa)-miR-450b-5p.** (**a**) representation of the nucleotide (nt) complementarity between the 22 nucleotide (nt) hsa-miR-450b-5p [highlighted in green; encoded at the miR-450 gene cluster at human chromosome (chr) Xq26.3 and nt position 134–155 of the AOAH mRNA 3′-UTR non-coding region (highlighted in yellow; encoded at human chr 7p14.2; the microRNA target prediction database (miRDB; https://mirdb.org/cgi-bin/search.cgi?searchType=miRNA&searchBox=hsa-miR-450b-5p&full=1; https://www.targetscan.org/cgi-bin/targetscan/vert_80/view_gene.cgi?rs=ENST00000538464.1&taxid=9606&members=&showcnc=0&shownc=0&showncf1=&showncf2=&subset=1) (both accessed on 28 January 2026) for miR-450b-5p and AOAH (NCBI Gene ID 313, https://www.ncbi.nlm.nih.gov/gene?Db=gene&Cmd=DetailsSearch&Term=313 (accessed on 8 January 2026)) indicated a very high miRNA-mRNA homology of 17/22, a target score of 77 (miR-450b and AOAH mRNA 3′-UTR are 77% homologous) and a strong 10 nt ‘seed’ sequence location at 145–154 nt of the AOAH 3′-UTR; (**b**) the ~300 nt AOAH 3′-UTR gene sequence; the inverted– red arrow indicates the start of the AOAH 3′-UTR non-coding sequence; last upward pointing red arrow is the end of the AOAH 3′-UTR; the yellow-highlighted sequence indicates the predicted miR-450b-5p binding site corresponding to the region shown in (**a**); (**c**) schematic diagram of AOAH mRNA-3′-UTR expression vector for luciferase reporter assay (plight Switch-3′-UTR: Cat#S810535; pLightSwitch^TM^ 3′-UTR Reporter GoClone^®^ Collection; Switchgear Genomics. Palo Alto CA, USA); in this vector an entire ~300 nucleotide AOAH 3′-UTR insert was ligated into the unique Nhe1-Xho1 site; there is no polyA tail included in the AOAH 3′-UTR insert; (**d**) HEK-293 cells transfected with AOAH-mRNA-3′-UTR expression vector luciferase reporter or an empty expression vector reporter as positive control were treated exogenously with a stabilized miR-450b-5p mimic (miR-450b-5p), or a non-targeting negative control miRNA sequence (miR-NC); see text for further details on all reagents and methods used in these experiments [12,24], compared to control cells, the AOAH 3′UTR vector exhibited decreased luciferase signal down to a mean of 0.48-fold of controls in the presence of 100 nM miR-450b-5p; this same vector exhibited no change in relative luciferase yield in the presence of control miR-NC; for each experiment, a luciferase signal was generated that included separate controls with each analysis; N = 3; * *p* < 0.05, ** *p* < 0.01 (one-way ANOVA), values represent mean ± standard error of mean (SEM); Microsoft Excel Analysis ToolPak, Excel for Microsoft 365 [23]; the results suggest a physiologically relevant miR-450b-AOAH-mRNA-3′-UTR interaction and a miR-450b-mediated down-regulation of AOAH expression (Figure 1). This pathogenic interaction may be related to the down-regulation of other immune, inflammatory, and synaptic system genes by upregulated miRNAs in the CNS and resulting in an overall deficit in protection against LPS-induced neurotoxicity [25].

Analytical tools, including TargetScan (release 7.0) and miRBase (release 22.2), identified a strong recognition feature and binding site for miR-450b-5p in the human AOAH 3′-UTR (Figure 2a). This binding region showed a 77.3% sequence homology (17/22 base pairs) with a calculated stability of −23.4 kcal/mol [22,23].

Transfection of a luciferase reporter vector containing the full 300 bp AOAH mRNA 3′-UTR (AOAH 3′UTR) (Figure 2b–d), together with stabilized miR-450b-5p mimic, resulted in a reduction in luciferase activity levels compared with those treated with an equivalent amount of non-targeting negative control of miRNA mimic (miR-NC). In addition, a dose-dependent effect was also observed with a reduction to 67% of control at 50 nM of miR-450b-5p and a further inhibition to 48% at 100 nM (Figure 2d). This result suggests miR-450b-5p-mediated downregulation of AOAH, similar to what is observed in AD brains. Meanwhile, transfection of empty luciferase reporter vector (control vector of the same reporter construct but without the 3′-UTR sequence, Empty 3′UTR) into HEK-293 cells, together with or without miR-450b-5p or miR-SC, caused no significant change in luciferase activity, confirming the specificity of miR-450b-5p for the AOAH 3′UTR. Interestingly, compared with Empty 3′UTR transfected control cells (without treatment with miR-NC or miR-450b-5p), control cells transfected with AOAH 3′UTR saw a slight but statistically significant reduction in luciferase activities, suggesting that the endogenous miR-450b-5p or other potential AOAH 3′UTR targeting miRNAs might mediate such effects.

The findings suggest a significant interaction between miR-450b-5p and the AOAH mRNA 3′-UTR, leading to reduced AOAH expression. This down-regulation may impair the inactivation of LPS, thereby promoting or sustaining LPS-mediated pro-inflammatory signaling as observed in the AD brain.

### 2.3. miR-450b-5p Suppresses AOAH Protein Expression in Human Neural Cells

**Figure 3:**
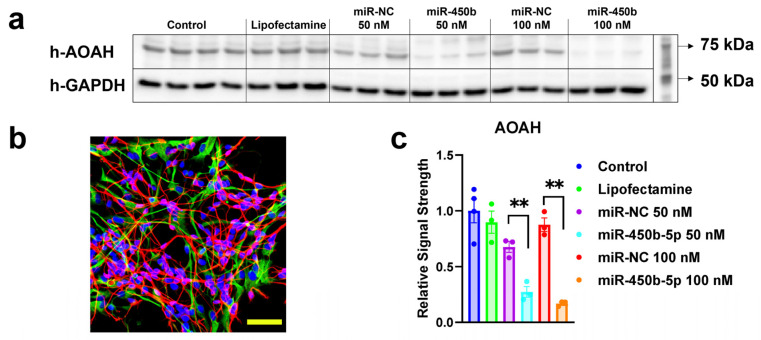
**miR-450b-5p suppresses AOAH protein expression in human neuronal–glial (HN) co-cultures.** (**a**) Representative Western blot showing h-AOAH protein levels in HN cells transfected with miR-450b-5p mimics (50 nM and 100 nM), compared to controls including Lipofectamine only, and scrambled negative control miRNA (miR-NC) at matching concentrations. GAPDH served as the loading control. (**b**) Immunofluorescence image of HNG cultures used in the experiment, showing staining of βIII-tubulin (red) as a marker for neurons, GFAP (green) as a marker for astrocytes, and DAPI (blue) as a marker for nuclei. Scale bar = 50 μm. (**c**) Densitometric quantification of AOAH signal intensities normalized to GAPDH and expressed relative to untreated control (set to 1.0). A dose-dependent reduction in AOAH expression was observed following transfection with miR-450b-5p mimics (** *p* < 0.01, one-way ANOVA with Tukey’s post hoc test, n = 3). Data are shown as mean ± SD with individual replicate values overlaid.

To determine whether miR-450b-5p directly impacts AOAH protein expression in human neural (HN) cells, we transfected primary HN cell cultures with either miR-450b-5p mimic or a non-targeting negative control (miR-NC) at two concentrations (50 nM and 100 nM). Western blot analysis (Figure 3a) revealed a marked and dose-dependent decrease in AOAH protein levels in cells treated with miR-450b-5p compared to all control groups, including lipofectamine-only, miR-NC 50 nM, and miR-NC 100 nM conditions. Quantification of band intensity normalized to GAPDH (Figure 3c) confirmed a statistically significant reduction in AOAH expression following miR-450b-5p treatment. Immunofluorescent imaging of the same culture (Figure 3b) confirmed the characteristic morphology and mixed neuronal–glial composition of the HN cells used in this experiment. Together, these results support the conclusion that elevated levels of miR-450b-5p are sufficient to suppress AOAH protein expression in human neural cells, consistent with a potential role for miR-450b-5p in promoting inflammatory vulnerability in the AD brain through AOAH down-regulation.

## 3. Discussion

### 3.1. miRNA Abundance, Speciation, and Complexity in AD Brain

miRNAs are a family of short (~22 nucleotide) non-coding single-stranded RNAs (ssRNAs) that play a central role in the post-transcriptional regulation of gene expression across diverse eukaryotic systems. This regulation is primarily mediated through nucleotide complementarity within the 3′-UTR of target mRNAs, leading to mRNA degradation and translational repression [26,27,28].

miRNAs are critical regulators of gene expression, influencing a variety of physiological and pathological processes. Their ability to modulate complex signaling networks enables miRNAs to contribute to the pathogenesis of numerous diseases in *Homo sapiens* [26,27,28,29].

As is the case for miR-450b-5p, specifically upregulated miRNAs have been associated with the down-regulation of AD-relevant target mRNAs, including those encoding neurofilament light chain (NF-L) filament protein, the triggering receptor expressed in myeloid cells (TREM2) and the post-synaptic cytoskeletal element SHANK3 gene expression [28,30,31]. Deficits in NF-L, TREM2, and SHANK3 may, in part, be responsible for, respectively, alterations in cytoarchitecture, deficits in the ability of microglia to remove cellular waste products and neurotransmission [28,29].

It should be noted that the HEK293 cell line used in our study reportedly exhibits modest levels of endogenous expression of miR-450b-5p, comparable to other microRNAs such as miR-9 and miR-34a [32]. Based on TargetScan predictions, several other miRNAs endogenously expressed in HEK293 cells, including miR-186-5p, miR-92a-3p, and miR-92b-3p, may also have potential binding sites within the AOAH 3′UTR. While these miRNAs could contribute minimally to baseline reporter signal, the strong and dose-dependent suppression of luciferase activity observed upon transfection with miR-450b-5p mimics indicates that the primary effect arises from the exogenously introduced miRNA rather than basal endogenous expression.

### 3.2. miR-450b-5p Activity in Human Disease

Human chromosome Xq26.3 encodes miR-450b-5p, a 22-nucleotide (nt), A+U-enriched, pro-inflammatory ssRNA. This miRNA is expressed in multiple cell and tissue types, including the brain, pituitary, and skeletal muscle, under both healthy and pathological conditions [33] (Figure 2a).

Overexpression of miR-450b-5p has been linked to various inflammatory diseases and pathological processes, including cancer progression [33], ischemic stroke [34], ischemia and reperfusion injury [20,30], hypoxia/re-oxygenation injury [35], major depressive disorder (MDD) [36,37], up-regulation of the pro-inflammatory transcription factor NF-kB (p50/p65) [38], AD (this report; Figure 1). Plasma exosomal miR-450b-5p was identified as a possible biomarker and therapeutic target for transient ischaemic attacks in rats [39]. Transcription of the human miR-450b-5p gene may itself be regulated by the pro-inflammatory transcription factor NF-kB (p50/p65) [40] (unpublished observations). This suggests a potential feedback loop in which NF-kB drives the expression of miR-450b-5p, amplifying pro-inflammatory signaling in both systemic and neurological disorders.

Interestingly, miR-450b-5p appears to have tissue- and context-dependent roles. In LPS-induced acute lung injury (ALI), for example, lncRNA MIR3142HG promotes inflammation by suppressing miR-450b-5p, leading to up-regulation of HMGB1 and worsening LPS-induced damage [41]. Similarly, circRNA circPalm2 sponges miR-450b-5p, indirectly promoting ROCK1 expression and aggravating inflammatory responses [42]. Inhibition of miR-450b-5p was found to ameliorate hepatic ischemia/reperfusion injury via targeting CRYAB [20], while in retina, exosomal miR-450b-5p secreted from exendin-4-stimulated endothelial cells showed protective effects on retinal ganglion cells against ischemia–reperfusion injury [30].

These observations suggest that miR-450b-5p function is highly dependent on the specific cellular and pathological context. Its injury-relieving role in certain tissues, such as the lung, and its pro-inflammatory effects in the liver highlight the complexity of its regulatory networks.

The involvement of miR-450b-5p with differing nature in these conditions highlights its pivotal yet complex role in the molecular mechanisms underlying inflammation and neurodegeneration, further implicating it as a potential therapeutic target for managing AD and other inflammatory diseases.

### 3.3. LPS and Acyloxyacyl Hydrolase (AOAH)

LPS, a key component of the outer membrane of Gram-negative bacteria, is the most potent activator of innate immunity and inflammasomes in *Homo sapiens* [25,31,43,44]. Key structural and functional features of LPS include: LPS consists of highly variable oligosaccharide/polysaccharide regions and a relatively conserved glycolipid region (lipid A); fatty acid acyl chains are attached to lipid A, forming the biologically active and endotoxic moieties [31,43]; these fatty acid chains drive extreme innate immune and inflammatory responses during host–pathogen interactions; a complete set of six acyl chains within the lipid A region is typically required for LPS detection by host pattern-recognition receptors [25,31,43,44].

AOAH (EC 3.1.1.77) is a 575-amino-acid carboxylic ester hydrolase that detoxifies LPS by removing secondary (acyloxyacyl-linked) fatty acids from the lipid A region, rendering LPS immunologically inert [25,31,43,44]. The enzymatic reaction catalyzed by AOAH is described as follows: 3-(acyloxy)acyl group of bacterial toxin + H20 = 3-hydroxyacyl group of bacterial toxin + a fatty acid (IUBMB Reaction ID: R05792).

AOAH has also been reported to possess diacylglycerol lipase, phospholipase, lysophospholipase, and acyltransferase activities in vitro [34,35,36]. Compartmentalized in intracellular membrane-bounded organelles, AOAH enables acyloxyacyl hydrolase and calcium ion binding activities involved in fatty acid metabolic processing and LPS degradation [15,16,17]. By degrading and neutralizing LPS, AOAH terminates the host response to bacterial infection and prevents prolonged and damaging inflammatory responses important for the recovery from a state of immune tolerance following infection by Gram-negative bacteria or LPS and other endotoxins originating from GI-tract microbiome sources [15,16,17]. A recent study showed AOAH-deficient mice had gut dysbiosis, increased levels of serum endotoxin, activation of microglia and pelvic allodynia, suggesting AOAH is a host determinant of normal gut microbiota and could potentially regulate pelvic pain through microglial modulation [19,45]. Furthermore, AOAH was found to reduce LPS-induced lipid accumulation in the liver of high-fat diet-fed mice, relieve hepatic inflammation, and minimize tissue damage [46]. Another study demonstrated that AOAH protects the brain from experimental stroke by regulating neutrophil-dependent BBB breakdown and cerebral infarction [47]. Interestingly, AOAH appears to have multiple ancillary physiological effects. For example, AOAH expressed in the hypothalamic paraventricular nucleus may be a novel genetic regulator of the inflammation- and stress-responsive corticotropin-releasing factor, a central regulator of the hypothalamic–pituitary–adrenal neuroendocrine axis [17].

### 3.4. Deficits in Gene Expression in AD

AD is a slow, irreversible, and progressive neurological disorder characterized by inflammatory neurodegeneration, the accumulation of amyloid beta (Aβ) peptides and tau proteins, and the permanent loss of neurons and synapses within the brain and CNS. Numerous studies employing advanced quantitative Northern analysis, RNA sequencing, and DNA array technologies have demonstrated a general reduction in gene expression within AD-affected brain tissues. This reduction is indexed by a significant decline in total mRNA abundance, reflecting the functional deterioration of brain cells and critical inter-neuronal signaling systems [29,48,49,50,51]. The reduction in mRNA populations in the brain and CNS is likely linked to an inability to meet homeostatic demands and/or a failure of molecular-genetic mechanisms required for proper cellular function [48,49]. Total mRNA population deficits have been significantly associated with the neurodegenerative processes observed in AD [48,49]. The observed downregulation of gene expression in AD appears, at least in part, to result from the up-regulation of specific miRNA species commonly found in AD-affected brains [48,49,50,52]. Many of these miRNAs are inducible and transcriptionally regulated by the pro-inflammatory NF-kB (p50/p65) transcription factor complex [14,24,29,50,51]. Additional pathogenic miRNAs and transcription factors may also contribute to this dysregulation [29,49,50,51]. Neurotoxins such as LPS have been observed to exploit Aβ peptides for cellular entry into neurons. Aβ peptide accumulation is a hallmark of AD neuropathology, and its facilitation of neurotoxic mechanisms highlights its dual role as both a biomarker and a pathogenic agent in AD [53,54].

These findings suggest a complex interplay of gene expression deficits, miRNA dysregulation, and neurotoxic mechanisms in the progression of AD, underscoring the multifaceted nature of the disease and its reliance on molecular-genetic interactions.

### 3.5. AOAH Activity in the Human Brain

A previous study comparing the mRNA abundance in human and murine brain cells shows that AOAH mRNA is more highly expressed in human than murine microglia [55]. The present studies demonstrate that AOAH expression is detectable in the temporal lobe neocortex of the human brain and in HNG cells. AOAH is expressed as a moderately abundant soluble mRNA but shows a significant reduction in AD-affected brain, with levels approximately 0.47-fold of those observed in age-, gender-, and post-mortem interval (PMI)-matched controls (Figure 1). The reduction in AOAH expression in AD brain tissue, potentially involving microglial populations, may impair the brain’s capacity for LPS detoxification. Furthermore, this reduced expression of AOAH may be linked to an increase in miR-450b-5p, a microRNA that targets the AOAH 3′-UTR and is a known inflammation regulator. This relationship was confirmed using a luciferase reporter assay with an AOAH-mRNA-3′-UTR expression vector, where the up-regulation of miR-450b-5p was associated with decreased AOAH activity (Figure 2). Furthermore, the targeting effect of miR-450b-5p on the AOAH gene was confirmed at protein levels in HNG cells in vitro (Figure 3). Since AOAH is a known detoxifier of LPS, the lack of AOAH in the AD brain may be expected to enhance or prolong LPS-associated neurotoxicity and thus contribute to pro-inflammatory signaling and progressive neurodegenerative changes within the AD-affected brain and CNS.

These findings highlight the pivotal role of AOAH in maintaining neuroimmune homeostasis and suggest that its dysregulation, mediated by miR-450b-5p, may be a critical factor in the pathogenesis of Alzheimer’s disease.

### 3.6. Limitations and Future Directions

While this study provides novel evidence linking reduced AOAH expression with increased miR-450b-5p activity in AD brain tissue, several methodological limitations should be acknowledged. First, the luciferase reporter assays were conducted in HEK293 cells rather than in neuronal or glial models. Although HEK293 cells are widely used for microRNA–target validation due to their high transfection efficiency, future studies employing human neuronal or microglial cells will be essential to confirm these regulatory effects in a more physiologically relevant context. Second, while our data from HNG cultures demonstrate that miR-450b-5p downregulates AOAH protein levels, the specific contribution of microglia, which represents a major cellular source of AOAH in the brain, remains to be investigated. Given the central role of microglia in LPS detoxification and neuroimmune regulation, future experiments using primary or induced pluripotent stem cell (iPSC)-derived microglia will be important to define the cell-specific mechanisms of miR-450b-5p-mediated AOAH suppression. Finally, in vivo studies in AD models will be necessary to assess how AOAH deficiency and miR-450b-5p overexpression interact to influence LPS-associated neuroinflammation and neurodegeneration.

## 4. Materials and Methods

### 4.1. Human Brain Tissues

Human brain tissues were sourced from the following institutions: Oregon Health Sciences Center (Portland, OR, USA); University of California Irvine Institute for Memory Impairments and Neurological Disorders (UCI MIND; Irvine, CA, USA); Louisiana State University Health Sciences Center–New Orleans (LSUHSC-NO) Neuroscience Center of Excellence (New Orleans, LA, USA); Archived RNA samples were also obtained from the University of Toronto (Toronto, ON, Canada). The use of these brain tissues adhered to the Institutional Review Board (IRB) and ethical guidelines at LSUHSC-NO and other donor institutions [21].

All neocortical tissue samples were derived from male or female Caucasian individuals, as the PMI, the time from death to brain-freezing, can influence RNA quality. To ensure RNA integrity, all tissues used in this study had a PMI of 4.1 h or less.

Samples were categorized based on the Center to Establish a Registry for Alzheimer’s Disease/National Institutes of Health (CERAO/NIH) criteria, following established guidelines. AD tissues used in these analyses were from patients with a clinical dementia rating of 2.0 to 3.0, indicative of moderate to severe stages of Alzheimer’s disease [56].

### 4.2. HEK-293 and HNG Cells in Primary Culture

HEK-293 cells (human embryonic kidney cells) (ATCC, Manassas, VA, USA) were cultured in Dulbecco’s Modified Eagle Medium (DMEM; Thermo Fisher Scientific, Waltham, MA, USA) supplemented with 10% fetal bovine serum (FBS; Gibco, Grand Island, NY, USA) and 1% penicillin–streptomycin (Thermo Fisher Scientific). Cells were maintained in a humidified incubator at 37 °C with 5% CO_2_ and passaged every 2–3 days to maintain exponential growth.

HNG cells, a co-culture of human neuronal and glial cells, differentiated from human neural progenitor cells (CC-2599; Lonza Biosciences-Cambrex, Walkersville, MD, USA), were maintained in human neural maintenance medium (HNMM). This medium was supplemented with human fibroblast growth factor, neuronal survival factor 1, epidermal growth factor, and gentamicin–amphotericin B (G/A), as previously described [21]. Notably, human primary neurons do not culture well in the absence of glial cells.

HNMM was refreshed at 3.5-day intervals. After two weeks of culture, HNG cells reach about 80% confluence, consisting of approximately 70% neurons and 30% astroglia; a good culture of human neurons requires the presence of astroglia for nutrition and support [52]. The cells were then treated with test miR-450b and control conditions at medium change [21].

### 4.3. Total RNA Isolation

To analyze the abundance of mRNA and miRNA of interest in human brain, neocortex tissues from two groups were selected: (i) AD group (N = 6); (ii) age-, gender-, and PMI-matched control group (N = 6). Total RNA, including miRNA, was isolated from human brain tissue samples using the mirVANA™ miRNA Isolation Kit (Cat. No. AM1561, Thermo Fisher Scientific) following the manufacturer’s protocol. Tissue samples (~10 mg) were homogenized in lysis buffer using a rotor-stator homogenizer (IKA Works, Wilmington, NC, USA). Purified RNA was quantified using a NanoDrop spectrophotometer (Thermo Fisher Scientific), and RNA integrity was assessed using an Agilent 2100 Bioanalyzer (Santa Clara, CA, USA).

### 4.4. mRNA Reverse Transcription and Quantitative PCR

For mRNA quantification, AOAH and β-actin mRNA levels were analyzed. Reverse transcription (RT) was performed using 1 µg of total RNA with the High-Capacity cDNA Reverse Transcription Kit (Thermo Fisher Scientific, Cat. No. 4368814) in a 20 µL reaction volume. The reaction mix contained random primers, RNase inhibitor, dNTPs, and reverse transcriptase. The RT protocol was as follows: 25 °C for 10 min, 37 °C for 120 min and 85 °C for 5 min.

Quantitative PCR (qPCR) was carried out using TaqMan™ Gene Expression Assays for AOAH (Assay ID: Hs01085536_m1) and β-actin (Assay ID: Hs01060665_g1) as the internal control. The reaction mix (20 µL) consisted of 1 µL cDNA, 1 µL TaqMan probe, and 18 µL of TaqMan™ Universal PCR Master Mix II (Cat. No. 4440040, Thermo Fisher Scientific). qPCR was performed on a Bio-Rad CFX96 Real-Time PCR System (Bio-Rad, Hercules, CA, USA) using the following cycling conditions: 50 °C for 2 min, 95 °C for 10 min, 40 cycles of 95 °C for 15 s and 60 °C for 60 s. Relative mRNA levels were calculated using the ΔΔCt method, normalized to β-actin mRNA levels.

### 4.5. miRNA Reverse Transcription and Quantitative PCR

For miRNA quantification, miR-450b-5p, miR-183, and U6 (as the internal control) levels were analyzed. Reverse transcription was performed separately for each miRNA using the TaqMan™ MicroRNA Reverse Transcription Kit (Cat. No. 4366596, Thermo Fisher Scientific) with specific stem-loop RT primers for each miRNA. Each 15 µL RT reaction contained: 10 ng total RNA, 3 µL of 5× RT primer, 1 µL of reverse transcriptase, 1.5 µL of 10× RT buffer, 0.15 µL dNTPs, 0.19 µL RNase inhibitor. The RT protocol was as follows: 16 °C for 30 min, 42 °C for 30 min, 85 °C for 5 min. qPCR was performed using TaqMan™ MicroRNA Assays for miR-450b-5p (Assay ID: 002207), miR-183 (Assay ID: 002269), and U6 (Assay ID: 001973) as the normalization control. The reaction mix (20 µL) included: 1.33 µL of RT product (cDNA), 10 µL of TaqMan™ Universal PCR Master Mix II, 1 µL of TaqMan miRNA-specific probe, and 7.67 µL nuclease-free water. qPCR conditions were as follows: 95 °C for 10 min, 40 cycles of 95 °C for 15 s and 60 °C for 60 s. All qPCR reactions were performed on a Bio-Rad CFX96 Real-Time PCR System.

### 4.6. Reporter Assay

#### 4.6.1. Reporter Vectors

The reporter assay utilized the pLightSwitch 3′UTR Luciferase Reporter Vector (SwitchGear Genomics, now Active Motif, Carlsbad, CA, USA) containing the AOAH 3′ UTR downstream of a RenSP luciferase reporter gene. Control experiments included an empty 3′UTR vector and a non-targeting control miRNA.

#### 4.6.2. Cell Transfection

HEK-293 cells were seeded into white, tissue culture-treated 96-well plates at a density sufficient to achieve 80% confluence by the time of transfection (typically 10,000 cells per well). Transfections were performed using the DharmaFECT Duo Transfection Reagent (Dharmacon, Lafayette, CO, USA, Cat. No. T-2010-02) according to the manufacturer’s protocol. Each transfection mix was prepared as follows: plasmid DNA: 100 ng of 3′UTR luciferase reporter vector (AOAH 3′UTR or empty control), LightSwitch miRNA mimic: Final concentrations of 50 or 100 nM for miR-450b-5p mimic or non-targeting negative control (miR-NC), DharmaFECT Duo Reagent: 0.15 µL per well, serum-free medium was added to a final volume of 20 µL per well for the transfection mixture. Cells were incubated with the transfection mixture for 24 h, after which the medium was replaced with 100 µL of pre-warmed, antibiotic-free complete medium.

#### 4.6.3. Luciferase Assay

At 48 h post-transfection, luciferase activity was measured using the LightSwitch Luciferase Assay Reagent (SwitchGear, Cat. No. LS010) as follows: add 100 µL of LightSwitch lysis buffer and substrate mix directly to each well; incubate the plate at room temperature for 30 min, protected from light; measure luminescence for 2 s per well using a SpectraMax M5 Microplate Reader (Molecular Devices, San Jose, CA, USA).

### 4.7. miRNA Mimic Transfection in Human Neural–Glial (HNG) Cells

Primary human neural–glial (HNG) cells were cultured for 2 weeks until reaching approximately 80% confluency in 12-well plates. Cells were then transfected with either hsa-miR-450b-5p mimic or a negative control (miR-NC) mimic at final concentrations of 50 nM or 100 nM, using Lipofectamine™ RNAiMAX Transfection Reagent (Thermo Fisher Scientific) or using an equivalent amount of transfection reagent alone following the manufacturer’s protocol. After 48 h of incubation, cells were lysed in M-PER™ Mammalian Protein Extraction Reagent (Thermo Fisher Scientific) supplemented with a 1× protease and phosphatase inhibitor cocktail (Sigma-Aldrich, St. Louis, MO, USA), and total protein was collected for Western blot analysis.

### 4.8. Western Blot Analysis

Protein concentrations from HNG cell lysate were determined using a BCA assay (ThermoFisher Scientific). For each sample, 20 µg of tissue lysate was resolved on a 4–12% Criterion XT Bis-Tris Gel (Bio-Rad) and subsequently transferred onto a polyvinylidene difluoride (PVDF) membrane (Bio-Rad). The membranes were blocked in SuperBlock™ (TBS) Blocking Buffer (ThermoFisher Scientific) for 1 h at room temperature. Membranes were then incubated overnight at 4 °C with primary antibodies specific to human AOAH (Proteintech, Rosemont, IL, USA) or GAPDH (Cell Signaling Technology, Danvers, MA, USA). After primary antibody incubation, the membranes were washed and then incubated with horseradish peroxidase (HRP)-conjugated secondary antibodies (Cell Signaling Technology) for 3 h at room temperature. Signal development was performed using SuperSignal™ West Dura Extended Duration Substrate (ThermoFisher Scientific), and the signals were visualized using an ImageQuant LAS 4000 imaging system (GE Healthcare, Chicago, IL, USA). Densitometry of immunoreactive bands was conducted using ImageQuant TL software Version 7.0 (GE Healthcare), and band intensities were normalized to beta-actin as a loading control.

### 4.9. Data and Statistical Analysis

Quantitative PCR (qPCR) data were analyzed using Bio-Rad CFX Maestro 2.3 Version 5.3.022.1030 (Bio-Rad), and relative expression levels of mRNA and miRNA were calculated using the 2^−ΔΔCt method, with β-actin (for mRNA) and U6 (for miRNA) as internal normalization controls. Luminescence values from reporter assays were normalized to non-targeting miRNA controls to evaluate knockdown efficiency. Protein levels were assessed by Western blot using GAPDH as the loading control. Band intensities were quantified by densitometry using ImageQuantTL software, and relative signal strength was normalized to the untreated or mock-transfected control group. All graphs, including scatter bar plots and boxplots, were generated using GraphPad Prism Software 10 Version 10.6.1 (892) (GraphPad Software, LLC, Boston, MA, USA). Data are presented as mean ± standard error of the mean (SEM). Statistical significance between groups was evaluated using one-way ANOVA followed by Tukey’s multiple comparison test, with a threshold for significance set at *p* < 0.05.

### 4.10. Ethics Statement

The culture of human brain tissue cells, acquisition, and handling procedures were conducted in strict adherence to the ethics review board policies of the donor institutions and the LSU Health Sciences Center (LSU-HSC). This work was approved by the Institutional Biosafety Committee/Institutional Review Board (IBC/IRB) at LSU-HSC, New Orleans, LA, USA.

## 5. Conclusions

LPSs are highly pro-inflammatory amphipathic glycoconjugates typically consisting of a hydrophobic lipid domain attached to a core oligosaccharide and a distal polysaccharide. A soluble enzyme found in endosomes or possibly lysosomes of cells such as macrophages and animal plasma, the acyloxyacyl hydrolase AOAH is known to terminate the host response to microbial-derived LPS via the deacylation of LPS [47,57]. LPS neutralization and inactivation prevent the prolonged, progressive and damaging pro-inflammatory signaling normally induced by intact LPS species. Different LPS species are generated by different Gram-negative bacilli and have high variability in their structure, activity, and pro-inflammatory potential [13,58]. Both LPS and a pro-inflammatory miR-450b-5p were found to reduce AOAH expression in HNG cells in primary co-culture. The abundance of AOAH was also found to be reduced in the AD brain, and miR-450b-5p was found to be significantly increased in age and gender-matched controls (Figure 1). The findings from in vitro transfection experiments suggest (i) that AOAH down-regulation is modulated by an increase in miR-450b-5p and a highly specific miR-450b-5p-AOAH mRNA-3′-UTR interaction (Figure 2) and (ii) that AOAH reduction and the AOAH-mediated inability to detoxify LPS may allow LPS to persist in AD-affected neural cells, where it promotes and sustains inflammatory signaling and progressive neurodegeneration. Therapeutic approaches involving strategically designed and stabilized miRNAs and/or anti-miRNAs (AMs; antagomirs) may be useful in the clinical management of AD and other complex neurological disorders in which LPS and specific microRNAs such as miR-450b-5p have been found to be increased, thus requiring an effective disease-modifying intervention.

## Data Availability

All data used in this review are publicly available and accessible on Medline (www.ncbi.nlm.nih.gov) and are indexed by the last names of the individual authors.

## Abbreviations

The following abbreviations are used in this manuscript:

LPS	Lipopolysaccharides
GI	Gastrointestinal
AD	Alzheimer’s disease
BBB	Blood–brain barrier
CNS	Central nervous system
mRNA	Messenger RNA
AOAH	Acyloxyacyl hydrolase
miRNA	MicroRNA
HNG	Human neuronal–glial
ROS	Reactive oxygen species
ssRNA	Single-stranded RNA
3′-UTR	3′ untranslated region
PMI	Post-mortem interval
NF-L	Neurofilament light chain
MDD	Major depressive disorder
